# Development and validation of the adaptive leadership behavior scale (ALBS)

**DOI:** 10.3389/fpsyg.2023.1149371

**Published:** 2023-09-27

**Authors:** Sophie Nöthel, Annika Nübold, Sjir Uitdewilligen, Jan Schepers, Ute Hülsheger

**Affiliations:** ^1^Faculty of Psychology and Neuroscience, Department of Work and Social Psychology, Maastricht University, Maastricht, Netherlands; ^2^Faculty of Psychology and Neuroscience, Department of Methodology and Statistics, Maastricht University, Maastricht, Netherlands

**Keywords:** adaptive leadership, adaptive leadership behavior scale, flexible leadership, dynamic environment, VUCA world, scale validation

## Abstract

Due to the rapid changes in today’s business world, leaders need to, more than ever, adequately and flexibly react to new and changing demands in the workplace. An instrument that captures adaptive leadership behavior is still missing, however. This study describes the development and validation of a concise and timely new leadership instrument, the Adaptive Leadership Behavior Scale (*ALBS*). Based on a thorough literature review, we developed 27 items as an initial item pool. We tested this set of items with leaders and followers in a pilot study to assess its relevancy and comprehensibility. In Study 1, a field study with 201 employees, we explored the internal structure of the initial item pool with a Principal Component Analysis (PCA). Based on the factor loadings resulting from a second PCA, we reduced the item pool, resulting in a 15-item scale for which we then assessed convergent and divergent validity. In Study 2, a field study with 311 employees, we replicated the findings of Study 1 and assessed additional convergent and divergent validity as well as the model fit with a Confirmatory Factor Analysis (CFA). In Study 3, a multi-source field study with 155 leader-follower dyads we replicated the CFA and additionally assessed criterion-related validity. Results show that the ALBS is a concise and valid instrument for assessing adaptive leadership behavior, thereby building the grounds to extend our understanding of antecedents, mechanisms and consequences of leadership in dynamic environments.

## Introduction

Today’s business world has changed rapidly. Exponential developments with regards to technology, digitalization and globalization provide an extremely challenging mix for organizations to stay competitive (Knights and McCabe, 2015; Uhl-Bien and Arena, 2017). Volatile, uncertain, complex and often ambiguous (in short: VUCA) circumstances require organizations to make decisions with a tremendous speed and to drive innovative business models (Uhl-Bien and Arena, 2017). Particularly in times of crisis, these trends are accelerated as shown by the current COVID-19 pandemic. But also, previous crises (e.g., the financial and oil crisis, the burst of the dot-com bubble, or trade wars due to increasing globalization) increased pressure on organizations worldwide to find ways to adapt to new situations and to stay competitive in the market.

Especially during VUCA times, leaders play a key role in organizations (Kok and Van den Heuvel, 2019). Their ability to adequately and flexibly react to new and changing demands in the workplace, known as adaptive leadership behavior, is strongly needed to ensure organizational functioning. Adaptive leadership incorporates a leader “changing behavior(s) in appropriate ways as the situation changes” (Yukl and Mahsud, 2010, p. 1). Although many scholars have acknowledged the importance of adaptive leadership behavior in the workplace (e.g., Adams et al., 2013; Corazzini et al., 2015; Hlalele et al., 2015; Preece, 2016; Mugisha and Berg, 2017), the concept still needs further refinement, tangibility and, most importantly, empirical scrutiny (Yukl and Mahsud, 2010). According to Yukl and Mahsud (2010, p. 81), “there is considerable ambiguity in the management and leadership literature about the nature of flexible leadership and how to assess it.” This critique is in line with the general call for more research on concrete leadership behaviors or ‘basic building blocks’ in order to come to a more nuanced theorizing and more actionable points for interventions in practice (Antonakis et al., 2011; van Quaquebeke and Epitropaki, 2018). Furthermore, existing instruments on adaptive behavior are not specific to *leadership* but rather focus on adaptive behavior in a broader sense, such as adaptive performance (e.g., Kröger and Staufenbiel, 2012) or individual adaptability (e.g., the I-Adapt Ployhart and Bliese, 2006). To address this gap, the current study presents a new, concise, tangible and behavior-oriented measure of adaptive leadership, the Adaptive Leadership Behavior Scale (ALBS).

Based on a thorough literature review, we provide a concrete and specific definition of adaptive leadership and present an instrument to measure adaptive leadership behavior. The instrument acknowledges four main aspects of adaptive leadership behavior: *accurately perceiving situational demands, maintaining a toolbox of behavioral strategies, balancing opposing demands and appropriately and flexibly applying these behaviors*. With three independent data sets, we validate this newly developed questionnaire to determine its psychometric properties as well as to provide evidence for construct and criterion-related validity. The availability of a new measure for adaptive leadership is important as it builds the ground for empirical research on the role and impact of adaptive leadership in organizations as well as for developing concrete action points for leadership programs and interventions.

The current paper contributes to leadership theory and practice in three important ways. First, by presenting a tangible, behavior-oriented measure of adaptive leadership, we answer the call for more research on concrete leadership *behaviors* rather than on abstract leadership *styles* (see e.g., van Quaquebeke and Epitropaki, 2018). By developing an instrument that targets concrete adaptive leadership behaviors that are key in VUCA environments, we contribute to the theoretical advancement of adaptive leadership theory in a meaningful way. So far, most previous work on adaptive leadership behavior is only theoretical and remains rather abstract, that is, specific leader behaviors have not been fully detailed yet (e.g., Yukl and Mahsud, 2010; Uhl-Bien and Arena, 2018). By presenting a concrete and straightforward measure to study adaptive leadership behavior in the field, we build the ground for future research and theory building on adaptive leadership. Second, by defining concrete aspects of adaptive leadership behavior, we also advance adaptive leadership theory and contribute to a better understanding of its potential nature and constituting aspects. By identifying and acknowledging four main aspects of adaptive leadership and testing its nomological network, we provide a clearer picture on the conceptual make-up of adaptive leadership and its constituting elements that contribute to the overarching construct. A better understanding of how adaptive leadership manifests in concrete behaviors and is related to convergent and divergent factors helps to advance conceptual clarity on a construct that has, to date, only been vaguely defined. Finally, by presenting a new behavior-oriented instrument of adaptive leadership and providing evidence on its criterion-related validity, we provide empirical evidence on its relevance for today’s workplaces. Being able to identify concrete adaptive leadership behaviors that are linked to beneficial organizational outcomes enables the creation of specific training interventions to help leaders widen their behavioral repertoire, help them to better identify the specific demands of different situations, strengthen their ability to flexibly react, and balance opposing demands in an appropriate way. In summary, this study helps organizations to make their leaders VUCA-capable (Sinar et al., 2014), thereby contributing to current and future organizational functioning in a meaningful way.

### Adaptive leadership behavior then and now

Adaptive leadership has been a topic of scholarly interest for the last decade (e.g., Yukl and Mahsud, 2010; DeRue, 2011; Doyle, 2017; Uhl-Bien and Arena, 2018). When taking a look back, research has come a long way from proposing a static, deterministic and top-down view of leadership to a more dynamic, interactive and developmental view. While in the 1940s, the trait approach to leadership dominated the field, proposing a list of traits that predict effective leadership behavior, in the 1970s, interest in situational theories of leadership, such as contingency theories, were of growing interest. Examples include the LPC Contingency Model (Fiedler, 1964), Path-Goal Theory (House and Mitchell, 1974) or Situational Leadership Theory (Hersey and Blanchard, 1977). Although these theories markedly advanced the field by acknowledging the importance of the situation, they have also been criticized for proposing a single optimal solution for a leader to act within a concrete situation. Critics argued for equifinality, stating that there can be more than one leadership behavior that is effective in a specific situation (McCall, 1977). Despite their promising propositions, interest in contingency and situational theories of leadership quickly declined as empirical support was lacking (Yukl, 2010). Amongst other reasons, this was because concrete and accurate measures that were needed to prove the theories’ assumptions were lacking and because many of the conducted studies relied on weak research designs (Korman and Tanofsky, 1975; Schriesheim and Kerr, 1977).

Another important aspect of adaptive leadership behavior is the acknowledgment of and reaction to different followers and their particular needs (Yukl and Mahsud, 2010). Although past leadership theories like transformational, servant or authentic leadership also acknowledge the role of followers (Yukl and Mahsud, 2010; Pant and Sinha, 2016), they have been criticized for failing “to capture the complexity of leadership processes in modern organizations” (Yukl and Mahsud, 2010, p. 83). For example, in today’s VUCA world, leadership behavior that aims to give concrete directions and convey an attractive vision of the future, as in transformational leadership, has only limited utility as it requires leaders to predict the future with a certain level of accuracy (Wanasika and Krahnke, 2018; Wong and Chan, 2018). Today’s constantly changing environment does not allow for this level of accuracy and rather calls for leadership behavior that continually adapts to the given circumstances and enables employees to cope with frequently changing situations (Heifetz et al., 2009). Thus, although previous leadership styles already consider interactions between leader and follower, they do not sufficiently consider the dynamics between situations, employees, and leaders’ behaviors and are therefore unsuitable for describing, understanding, and advancing leadership in a VUCA environment (Wheatley and Frieze, 2010; Uhl-Bien and Arena, 2018; Wanasika and Krahnke, 2018).

In recent years, calls for new ways of leading that capture these dynamics have increased accordingly. Although the key objectives of effective leadership remain the same, e.g., to motivate followers to reach organizational goals, several scholars argued for the need to define leadership processes differently (Yukl and Mahsud, 2010; DeRue, 2011; Uhl-Bien and Arena, 2017; Wanasika and Krahnke, 2018). The most popular theory within this approach is Complexity Leadership Theory (Uhl-Bien and Arena, 2017, 2018). The theory conceptualizes leadership as a complex, interactive, dynamic system that enables employees to work, interact and connect with each other in ways that enable innovation, learning and novelty. Despite the value of these approaches and although we draw upon their idea that leadership should be viewed as a dynamic and adaptive process that accounts for the complexity in organizations (e.g., DeRue, 2011), we question their tangibility and utility for empirical research in their current form. For example, Complexity Leadership Theory proposes that leadership emerges from synergies between individual and collective interactions in a self-organizing system (Uhl-Bien and Arena, 2018; Wanasika and Krahnke, 2018), but it stays unclear what synergies between individual and collective interactions actually look like and how we can measure them, how leadership itself emerges and how all this translates into concrete behaviors. Unsurprisingly, empirical support for these complex and rather vague theoretical assumptions is still lacking (Tourish, 2019). Without a clearly defined construct and a common approach, leadership seems to become everything and nothing (DeRue, 2011, p. 131). As also in complex systems, formal leaders are part of today’s organizational structures, we explicitly only focus on concrete adaptive leadership behaviors as tangible, measurable, but yet central part of the aforementioned complex approaches. Adaptive leaders need to be able to adjust their behaviors flexibly to the situation, such as monitoring internal and external dynamics, deciding when to make strategic changes, relinquishing authority to others when required as well as being sensitive to the needs of subordinates (Yukl and Mahsud, 2010).

Building on previous models of adaptive leadership (e.g., Yukl and Mahsud, 2010), we propose that adaptive leadership behavior incorporates four main aspects: *accurately perceiving situational demands, maintaining a toolbox of behavioral strategies, balancing opposing demands and appropriately and flexibly applying these behaviors*. These four aspects are proposed to be equally relevant and necessary for adaptive leadership behavior, with some being more implicit (e.g., perceiving situational demands) but still equally relevant for adaptive leadership behavior. In the following, we describe how these four aspects collectively contribute to the holistic concept of adaptive leadership behavior.

**FIGURE 1:**
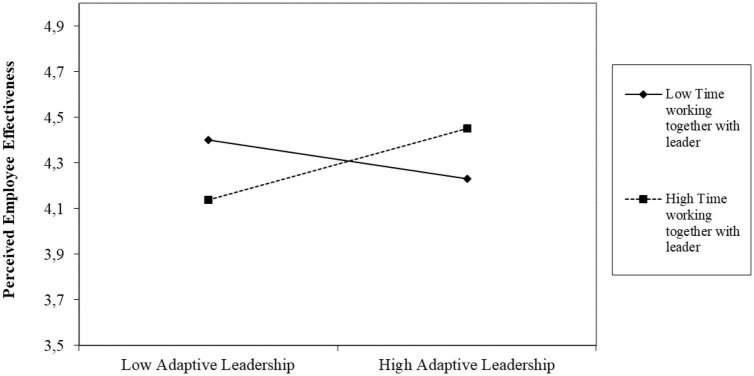
Analysis of Hypothesis 5c. Figure illustrates interaction effect of time working together and adaptive leadership on leaders’ perceived employee effectiveness.

Adaptive leaders need to be able to recognize adaptive pressures, that is, to understand situational demands (e.g., follower’s needs or environmental demands) in order to adjust their behavior accordingly (Kaplan and Kaiser, 2003; Baron et al., 2018). Accurately perceiving situational demands is important in order to correctly identify the relevant situational cues, such as different needs of customers and followers, and use them as informative basis for further action. This enables leaders to anticipate what is needed in a specific situation and how to appropriately react to it (Ployhart and Bliese, 2006). Situations may entail different types of challenges, for example, technical and adaptive challenges. Technical challenges or problems can be solved by existing expertise and by using rather traditional methods and organizational processes (e.g., if a production machine stops working, you can call a technical expert to fix the problem). When faced with adaptive challenges, such as unknown or not clearly defined problems, leaders cannot simply draw on prior knowledge, but need to come up with a new approach to solve the problem (Wong and Chan, 2018). Neither of the two types of challenges is easier to solve but they need to be tackled differently. In case of adaptive challenges, the most appropriate behavior varies from situation to situation. It could range from stepping back and letting the team take the lead (e.g., in the sense of shared leadership) to directing the team when no one knows how to proceed, or to balance opposing demands simultaneously (Wong and Chan, 2018). For both types of challenges, an accurate situational assessment also helps leaders to understand what their followers or stakeholders need so that the applied behavioral strategies become successful (Yukl and Mahsud, 2010; Figure 1).

When leaders have assessed the demands of a specific situation, they have to decide how to react to them. For an adequate reaction, maintaining a variety of behavioral strategies from which the leader can chose is vital (Ployhart and Bliese, 2006). The broader the behavioral repertoire of leaders, the better they are able to select the best-fitting behavior to the situation at hand. Again, these behaviors can range from taking over control if needed (i.e., authoritarian leadership) to relinquishing authority to others when required (i.e., participative leadership). Other examples of behaviors could be to initiate change, to apply an active coping style when change occurs or to provide a vision on how to deal with changing requirements in the future. Thus, an adaptive leader has the option to choose from a variety of leadership behaviors and can potentially combine them in a way that it benefits the situation (Ployhart and Bliese, 2006).

Particularly in situations where the leader is confronted with opposing demands, the ability to balance those conflicting requirements is important for an appropriate behavioral reaction. In dynamic and complex environments, situations are often ambiguous and not easily solved by one clear cut solution. Instead, situational demands may seem incompatible, requiring an adaptive leader to somehow balance those opposing demands. Balancing opposing demands thus means to accept and acknowledge incompatible demands in order to react to them appropriately. An organization’s long-term success increasingly depends on the capability of addressing and integrating opposing demands at the same time (Smith and Tracey, 2016). For example, leaders need to balance short term and long term strategies that seem conflicting but are both equally relevant for reaching long-term success (e.g., Slawinski and Bansal, 2017), such as investing in innovations while making sure that the core business stays profitable (Svahn et al., 2017). Thus, both demands are important for an organization’s survival and consequently, leaders need to balance them effectively. This notion resembles the idea of paradoxical leadership behavior which is defined as “leader behaviors that are seemingly competing, yet interrelated, to meet competing workplace demands simultaneously and over time” (Zhang et al., 2015, p. 539). Previous research has evidenced that paradoxical leadership behavior is positively related to adaptive behavior (Zhang et al., 2015).

Ultimately, for adaptive leadership behavior to become successful, leaders have to apply the behaviors from their behavioral repertoire flexibly and appropriately. For this, adaptive leaders draw on all the aforementioned behaviors: accurately assessing the adaptive pressures of a situation helps leaders to understand the specific requirements of a certain situation. By maintaining a wide variety of behavioral strategies as well as by balancing opposing demands, they can select and apply the most appropriate behavior for the assessed situation which finally results in successful adaptive leadership behavior (Ployhart and Bliese, 2006; Yukl and Mahsud, 2010).

In summary, by truly understanding the situation at hand and by being able to selectively apply a broad variety of (opposing) behaviors, adaptive leaders have the necessary skills to respond in a flexible and appropriate manner (Yukl and Mahsud, 2010). By flexibly adjusting their behavior according to the situation and necessities at hand, adaptive leaders are able to orchestrate their team through volatile, uncertain, complex and ambiguous times.

### Overview of studies

In this paper, we present a concise, tangible, behavior-oriented instrument of adaptive leadership. Specifically, after generating an initial pool of items, we conducted a pilot study to verify whether our items are clear, comprehensible and relevant to our target group (i.e., leaders and followers). Then, in Study 1, we conducted a cross-sectional field study with 201 followers to explore the internal structure of our item pool with an Principal Component Analysis (PCA) and assessed the construct validity (i.e., convergent and divergent validity) of our instrument. In Study 2, a cross-sectional field study with 311 followers, we first tested whether we can replicate the internal structure of the instrument with an PCA. Additionally, we extended the test of the nomological network of adaptive leadership by including additional convergent and divergent constructs and assessed model fit with a Confirmatory Factor Analysis (CFA). In Study 3, a cross-sectional multi-source field study with 155 leader-follower dyads, we again aimed to replicate the internal factor structure of our instrument with a CFA and additionally assessed its criterion-related validity.

### Measure development and item generation

To generate items for the Adaptive Leadership Behavior Scale, we followed the procedure recommended by Hinkin (1998). First, we thoroughly reviewed the literature to establish a theoretically sound and comprehensive definition of adaptive leadership behaviors (Hinkin, 1998). We reviewed articles dealing with adaptive leadership or related constructs such as flexible or agile leadership (e.g., Uhl-Bien and Marion, 2009; Hannah and Avolio, 2010; Yukl and Mahsud, 2010; DeRue, 2011; Head and Alford, 2015). Items were generated deductively by deriving short and simple statements that adequately represent the construct of adaptive leadership, including the four behaviors of accurately assessing situational needs, maintaining a toolbox of behavioral strategies, balancing of opposing demands and applying these behaviors appropriately and flexibly. Based on the literature review, an initial pool of 27 items was generated to assess adaptive leadership behavior as means to effectively lead in today’s VUCA world. For each of the four behaviors, we generated four to nine items. For perceiving situational demands, we developed six items (e.g., “My supervisor quickly grasps what kind of leadership behavior is optimal for a specific situation”). We propose that leaders are aware of situational requirements, are able to “read” situations and can draw meaningful conclusions from them. For maintaining a wide variety of behavioral strategies, eight items were developed that measured the extent of a leader’s behavioral repertoire and the behavioral options he/she has to react to different situations, employees or tasks. A sample item is “My supervisor possesses a wide variety of leadership behaviors he/she can selectively apply.” For the third behavior that emerged from the literature review, balancing of opposing demands, we developed four items. It entails the ability to balance and integrate divers or even opposing requirements as well as the ability to take different perspectives into account. A sample item is “My supervisor is able to balance opposite types of behavior (e.g., controlling vs. empowering) in a way that is appropriate for the situation.” For the appropriate and flexible application of behavior, we developed nine items. This behavior relates to the leaders’ ability to flexibly change behaviors and strategies according to the situation at hand rather than applying the same leadership style to any given situation, regardless of how appropriate it is. A sample item is “My supervisor adjusts his or her leadership behaviors to the demands of the specific situation.” For all four aspects of adaptive leadership, reversed-scored items were included to prevent response biases. Reversed-scored items improve scale validity by urging respondents to read the respective items more slowly and carefully before selecting a response (Józsa and Morgan, 2017). The usefulness of reversed-keyed items has been discussed controversially in the past as they can lead to method effects (Motl and Distefano, 2002). Weijters et al. (2013, p. 333) argue, however, that although method effects might occur, “it is better to be aware of them and to be able to take corrective action rather than to ignore them completely.”

### Pilot test

After generating the initial pool of items, we tested (a) the comprehensibility and (b) the relevance of the developed items with a relevant target group (i.e., both leaders and followers) to check whether they understood the items well and found them representative of the construct. After introducing the study’s objective and definition of adaptive leadership behavior personally to the participants, they were given access to an online questionnaire featuring the initial item set. Three leaders and five followers rated the items on the two criteria on a 5-point Likert-type scale and were also asked to freely comment on the items. Two of the three leaders were male, one was female. They were between 30 and 50 years old with different levels of leadership experience. One of them had already 25 years of leadership experience, while the other two had 2–4 years of leadership experience. On average, the items were rated as very comprehensible (*M* = 4.0, *SD* = 1.4) and relevant (*M* = 4.5, *SD* = 0.9). In addition, five followers rated the questionnaire on both comprehensibility and relevance. Four of them were female, one was male and their age was between 27 and 35 years. They were in an active employment relationship for 2–5 years. On average, the items were rated as comprehensible (*M* = 3.8, *SD* = 1.4) and relevant (*M* = 3.8, *SD* = 1.3) for the described purpose. A few participants stated that some items were not easy to understand. We noticed that whenever items received slightly lower relevance ratings, these scores consistently appeared in combination with a reduced comprehensibility rating. Therefore, we decided to still include these items (partly with adjusted wording) in the validation study for empirical testing. Hence, we reworded four of the 27 items, including two reversed items, to make them more comprehensible.

## Study 1

All studies were approved by the ethical committee of the authors’ home university (code to be depicted after publication). In the first study, we explored the internal structure of our initial item pool with a Principal Component Analysis (PCA) and assessed convergent and divergent validity to test the nomological network of the developed instrument. To assess convergent and divergent validity, we identified other constructs that were expected to be substantially related to adaptive leadership behavior (convergent validity), and constructs that were expected not to relate to adaptive leadership (divergent validity). We only chose measures with good psychometric properties that are well established in the literature (Bühner, 2011). For testing convergent validity, we included cognitive flexibility and emotional intelligence in our survey. Cognitive flexibility includes “a person’s (a) awareness that in any given situation there are options and alternatives available, (b) willingness to be flexible and adapt to the situation, and (c) self-efficacy in being flexible” (Martin and Rubin, 1995, p. 623). Previous research has already shown a positive relationship between individuals’ adaptability and their cognitive flexibility (Hamtiaux and Houssemand, 2012). In line with this, we propose that cognitive flexibility and adaptive leadership behavior are positively and strongly related as acting and leading in an adaptive way is not possible without flexibility in thinking. Furthermore, emotional intelligence should show strong positive correlations with adaptive leadership behavior. Emotional intelligence is defined as “the subset of social intelligence that involves the ability to monitor one’s own and others’ feelings and emotions, to discriminate among them and to use this information to guide one’s thinking and actions” (Salovey and Mayer, 1990, p. 189). It incorporates four dimensions: self-emotions appraisal (SEA), others-emotions appraisal (OEA), regulation of emotion (ROE) and use of emotion (UOE) (Law et al., 2004). We expect a conceptual overlap between the two sub-dimensions of emotional intelligence that target the appraisal and management of others’ emotions (i.e., OEA and ROE) and adaptive leadership behavior because adaptive leaders need to have a high awareness of their followers’ emotions to adequately react to their needs (Doyle, 2017). Without being sensitive to the emotions and needs of others, adaptive leaders will not be able to switch perspectives and use this information to adapt their behavior in an adequate way. In sum, we propose:

*H1: Adaptive leadership behavior shows positive correlations to the convergent constructs (a) cognitive flexibility and (b) emotional intelligence.*

For divergent validity, we included rigidity in the survey. Rigidity can be regarded as a construct opposite to adaptive leadership behavior as it is defined as “the tendency to develop and perseverate in particular cognitive or behavioral patterns, and such patterns being continuously employed in situations where the pattern is no longer effective” (Morris and Mansell, 2018, p. 3). Rigid persons are unable to deal with unstructured, unpredictable and complex situations where no clear or previously known solution can be applied. Hence, adaptation of behavior to frequently changing situations is not a behavior that rigid leaders would be able to exhibit (Steinmetz et al., 2011). Previous research has already shown that individual adaptability has a negative relationship with rigidity (Hamtiaux and Houssemand, 2012). Therefore, we expect higher levels of adaptive leadership behavior to be associated with lower levels of rigidity. In sum, this leads to the following hypothesis:

*H2: Adaptive leadership behavior is negatively related to rigidity.*

### Method

#### Sample and procedure

Data was collected using Prolific,^1^ an online data collection platform. Recruitment via data collection services has been shown to be as representative and at least as reliable as data collection via more traditional methods such as standard internet samples (Paolacci and Chandler, 2014; Buhrmester et al., 2016). Participation requirements included being in an active employment relationship (full- or part-time), having a direct supervisor, and being fluent in English. On average, respondents needed 8 min to complete the questionnaire. As an incentive, participants received 0.99 pounds for their participation. Participation was voluntary and respondents were allowed to stop participation at any time. In total, 201 participants completed the study, 135 females and 66 males. The sample size is in line with the recommendation of Hinkin (1998) for scale development of having at least 150 respondents. Most respondents were either between 26 and 34 years (44.8%) or between 36 and 45 years old (23.4%). Half of the participants (49.3%) worked for their current supervisor for 1–3 years, 19.9% for less than a year, 13.9% for 4–6 years and 16.9% for more than 6 years.

#### Measures

All items in this study were formulated in English and all response scales ranged from 1 (s*trongly disagree*) to 5 (*strongly agree*). The initial pool of 27 items was administered to measure adaptive leadership behavior. To assess construct validity, we used well established scales. If needed, we slightly adapted the selected scales to the business context so that all of them focused on the supervisor’s behavior. To measure cognitive flexibility, we used the 12-item Cognitive Flexibility Scale by Martin and Rubin (1995). A sample item is “I have the feeling that my supervisor is willing to work at creative solutions to problems.” Cronbach’s alpha was 0.89, 95% CI [0.87, 0.91] The two sub-scales of emotional intelligence others-emotions appraisal (OEA) and regulation of emotion (ROE) were assessed with the Wong and Law Emotional Intelligence Scale (WLEIS) (Wong and Law, 2002). A sample item for OEA is “My supervisor is sensitive to the feelings and emotions of others.” A sample item for ROE is “My supervisor is able to control his/her temper so that he/she can handle difficulties rationally.” Cronbach’s Alpha for the two combined sub-scales was 0.94, 95% CI [0.92, 0.95].

To assess divergent validity, we used the 10 rigidity items of the CAT-PD-SF scale (v1.1) by Simms et al. (2011). A sample item of rigidity is “My supervisor finds it difficult to consider valid opinions that differ from his/her own.” Cronbach’s Alpha was 0.96, 95% CI [0.95, 0.97].

### Results

To analyze the internal structure of the developed questionnaire, a Principal Component Analysis (PCA) with Oblimin Rotation was performed using SPSS version 25. The goal of a PCA is to explain the variance-covariance matrix of the observed variables by a smaller number of factors/components in order to describe and understand the relationships and underlying processes among observed variables (Tabachnick and Fidell, 2013). For our research purposes, we chose PCA in which no structure is imposed as this seemed most suitable when developing a new instrument.

First, and prior to conducting the PCA, we checked if the data was suitable for factor analysis. The correlation matrix produced many coefficients of 0.30 and above. This is a good indication that the sample is suitable for factor analysis because if correlations are too low (e.g., less than 0.30), variables are not sufficiently associated for the extraction of common factors (Tabachnick and Fidell, 2013). The Kaiser-Meyer-Olkin (KMO) value is an additional source to determine if a data set is factorable; it should be higher than the recommended value of 0.60 (Kaiser, 1970, 1974). The KMO value for this data set was 0.96 and hence factor analysis should be appropriate to extract distinct and reliable factors (Tabachnick and Fidell, 2013). Also, Bartlett’s Test of Sphericity (Bartlett, 1954) supported the suitability of the data set for factor analysis by reaching statistical significance (*p* < 0.001).

In a second step, we started to extract the factors using the raw item scores as we conducted the PCA based on the correlation matrix. The aim of PCA is to use as few factors as possible to describe the variance-covariance matrix of the observed variables. To define how many factors should be extracted, a combination of Kaiser’s K1 rule, scree plot and parallel analysis was used. The Kaiser’s K1 rule states that only factors with an eigenvalue of 1.0 or higher are retained to represent the data set with the least number of factors necessary (Tabachnick and Fidell, 2013). The PCA revealed a dominant first eigenvalue of 15.78 and two minor secondary factors with eigenvalues slightly higher than one (1.52 and 1.04), indicating a three-factor solution. As this technique may result in the extraction of too many factors (Pallant, 2013; Wood et al., 2015), we proceeded with the two additional tests. The scree plot confirmed the dominant first eigenvalue by a clear change of slope after the first component followed by a flat curve, indicating a one-factor solution. In addition, we ran the parallel analysis by Horn (1965), testing the probability that a factor is due to chance (Wood et al., 2015), which led to acceptance of the first factor. As parallel analysis is one of the most accurate approaches in identifying the adequate number of factors (Zwick and Velicer, 1986; Hubbard and Allen, 1987; Bühner, 2011; Wood et al., 2015) and the scree plot also confirmed this result while the dominant eigenvalue also pointed in this direction, we decided to retain one factor. Therefore, following the recommendations by Pallant (2013), we repeated the PCA with one fixed factor only instead of random factors. This one-factor solution explained 58.44% of the variance. Due to the one-factor solution, Oblimin rotation could not be applied.

Based on the results of this second PCA with one fixed factor, we checked whether the length of the scale could be shortened to minimize response biases caused by boredom and fatigue (Schriesheim and Eisenbach, 1990), to maximize parsimony (Thurstone, 1947), and to create an economic scale. To reduce the initial 27 items, we selected items based on a combination of criteria, such as their factor loadings being equal to or over 0.80 (Yong and Pearce, 2013), their clarity and comprehensibility, and (across items) their ability to cover the content breadth of adaptive leadership behavior [each dimension is covered with one (maintaining a toolbox of behavioral strategies) to six (appropriate and flexible application) items], resulting in a selection of 15 items (see Table 1).

**TABLE 1:** Results of Principal Component Analysis Study 1: Adaptive Leadership Behavior Scale (ALBS).

ALBS item	Component 1
**Maintaining a toolbox of behavioral strategies**	
1. My supervisor’s leadership behavior varies in an appropriate way depending on the task.	0.51
2. My supervisor’s leadership behavior varies in an appropriate way depending on the subordinate.	0.43
3. My supervisor possesses a wide variety of leadership behaviors he/she can selectively apply.	0.75
4. My supervisor is not able to use a variety of complimentary behaviors (e.g., taking control but also sharing responsibilities).	0.54
**5. My supervisor is able to focus on and manage the task at hand while keeping an eye on employee’s needs.**	**0.81**
**6.** My supervisor knows how to support shared leadership, where leadership responsibility is evenly distributed among team members, whenever the situation calls for it.	0.79
**Accurately perceiving situational demands**	
**7. My supervisor quickly grasps what kind of leadership behavior is optimal for a specific situation.**	**0.81**
**8. My supervisor realizes when his/her leadership style should change due to changes in the situation.**	**0.82**
9. My supervisor often fails to recognize that his/her leadership behavior is not optimal for the situation at hand.	0.70
10. My supervisor does not adjust his/her leadership style if the external environment requires him/her to do so.	0.77
**11. My supervisor tries to understand the needs of his/her subordinates and adjusts his/her responses in a fitting way.**	**0.81**
**12. My supervisor is able to continuously adjust his/her behavior to the right degree to the circumstances at hand.**	**0.88**
**13. My supervisor recognizes changes in task priorities and the need to modify his or her leadership behavior.**	**0.83**
14. My supervisor does not recognize when shared (i.e., team leadership) instead of heroic leadership (i.e., by him/herself alone) is required.	0.63
**Appropriate and flexible application**	
**15. My supervisor reacts to unforeseen circumstances or problems with an appropriate response.**	**0.81**
16. My supervisor is not able to behave in an adaptive way when confronted with changing conditions that require a change in strategies/behaviors.	0.80
**17. My supervisor adjusts his or her leadership behaviors to the demands of the specific situation**	**0.84**
18. My supervisor rigidly uses one specific leadership style independent of changes in the Situation.	0.57
19. My supervisor is not able to provide direction to his/her subordinates in complex situations where no clear solutions exist.	0.75
**20. My supervisor adapts his or her leadership behavior when unexpected events occur.**	**0.85**
**21. My supervisor is capable of adjusting his/her leadership style based on the needs of his/her subordinates.**	**0.90**
**22. My supervisor stays focused on the goal while remaining flexible in what leadership approaches, he/she uses to achieve the goal.**	**0.86**
**23. My supervisor easily switches between directive and shared leadership according to the actual situation.**	**0.80**
**Balancing opposing demands**	
**24. My supervisor is able to balance opposite types of behavior (e.g., controlling vs. empowering) in a way that is appropriate for the situation.**	**0.87**
**25. My supervisor is able to lead through difficulties, ambiguity and complexity.**	**0.85**
**26. My supervisor is able to balance various conflicting needs of different stakeholders.**	**0.83**
27. My supervisor is not able to shift perspectives and view things from different angles.	0.56

*N* = 201. Results of Principle Component Analysis of initial 27 items. 15 retained items are marked in bold lettering.

#### Construct validity

In line with Hypothesis 1, both convergent constructs, cognitive flexibility (*r* = 0.88, *p* < 0.001) and emotional intelligence (*r* = 0.81, *p* < 0.001), were strongly positively related to adaptive leadership behavior. Hypothesis 2 was also confirmed as rigidity was strongly negatively related to adaptive leadership behavior (*r* = −0.68, *p* < 0.001).

Taken together, the results of Study 1 revealed a clear one-factor solution of the ALBS with good psychometric properties. Strong positive relationships with convergent constructs as well as a strong negative relationship with a divergent construct indicate a high degree of construct validity. To confirm the factor structure and further test its psychometric properties and construct validity, we tested the 15-item ALBS in a second sample in Study 2.

#### Common method bias

As with all data coming from the same method, there is the potential for the occurrence of common method biases. This means the estimated strength of relationships among the constructs of interest might be inflated in a systematic way due to sharing the same method (i.e., a self-report survey) (Podsakoff et al., 2003). Therefore, we followed the recommendation of Podsakoff et al. (2003) to control for common method bias in a statistical way (see Supplementary Table 2). We used Mplus to model and control for a method factor in addition to our variables of interest. For doing this, we fitted a model with three factors. The first factor represented adaptive leadership behavior. The second factor represented a construct which does not play a role in the research question of Study 1, but was collected with the same methodology (i.e., authentic leadership). This was done to isolate the potential bias caused by the method itself rather than the content of the variables involved. Most importantly, however, the items of both constructs can be suspected to be susceptible to the same method bias. In addition, we created a third factor, the method factor, letting all items load on this factor. We allowed the two construct factors to correlate but neither of them was allowed to correlate with the method factor. Finally, we regressed the first factor, representing adaptive leadership behavior, on the convergent or divergent construct which was proposed to correlate with adaptive leadership behavior. With this approach, it is possible to remove common method bias from the relationship of interest (Podsakoff et al., 2003). The standardized model results showed that both cognitive flexibility (estimate = 0.66, *p* < 0.001) and emotional intelligence (estimate = 0.49, *p* < 0.001) as well as rigidity (estimate = −0.21, *p* = 0.001) remained significantly related to adaptive leadership behavior after controlling for common method bias.

## Study 2

While the aim of Study 1 was to explore the internal structure of the initial item pool, to reduce items, as well as to assess convergent and divergent validity, the aim of Study 2 was to confirm the factor structure with a PCA and to analyze additional convergent and divergent constructs to extend the test of the nomological network. In a second step, we tested the model fit of the one-factor model structure resulting from the PCA by means of a CFA. Here, we included the final one-factor model to analyze the model fit.

In addition to cognitive flexibility and emotional intelligence, we now tested three leadership constructs that we expect to be conceptually related to adaptive leadership behavior (e.g., authentic leadership, transformational leadership and servant leadership). Authentic leadership is defined by Walumbwa et al. (2008, p. 94) as leadership behavior that uses and promotes positive psychological skills as well as a positive ethical climate. Based on that, authentic leaders promote greater *self-awareness*, an *internalized moral perspective*, a *balanced processing* of information and *relational transparency* when working with followers in order to foster positive self-development. Transformational leadership is a leadership approach where the leader aims to transform and motivate followers by providing an inspiring vision of the company and encourages employees to look beyond their individual interest in order to contribute to the greater good and mission of the organization. A transformational leader challenges individual assumptions but also acts as mentor or coach to followers (Bass, 1990). According to Bass and Avolio (1994), transformational leadership consists of the following four dimensions: *idealized influence*, *inspirational motivation*, *intellectual stimulation*, *individual consideration.* Servant leaders are characterized by putting their own interests and needs behind those of their followers in order to support them to pursue a successful career (Greenleaf, 2007). We expect the three leadership styles to be positively related to adaptive leadership behavior as they share the idea that acting in line with follower’s needs is important for their development, job performance and motivation. Even though these leadership styles do not focus explicitly on adaptive leadership behavior, all of them acknowledge the importance for leaders to adapt their behavior to some extent and to consider followers’ perspectives in order to lead successfully (e.g., Ilies et al., 2005; Wang et al., 2017; Williams et al., 2017). However, adaptive leadership behavior also differs in that it not only includes reacting to follower’s needs but also to situational demands. Only in combination, these two main aspects make adaptive leadership behavior successful. In sum, we hypothesize the following:

H3: *Adaptive leadership behavior shows high construct validity by showing positive relations to the convergent constructs (a) authentic leadership, (b) transformational leadership and (c) servant leadership.*

To assess divergent validity, we included two leadership styles, laissez-fair leadership and directive leadership, in addition to rigidity. In laissez-fair leadership, leaders do not interact with followers, avoid making decisions, and refrain from providing followers with feedback or rewards (Antonakis et al., 2003). Followers’ needs are neither recognized nor satisfied (Skogstad et al., 2007). This is not in line with adaptive leadership behavior where variability in leadership behavior and interactions between followers and leaders stand central (DeRue, 2011). Since not leading at all may, in some situations, still be adaptive, we, however do not expect a negative relationship between the two constructs, but rather a weak one. Directive leadership mainly includes that leaders use their position power to instruct their followers, give them commands and assign goals without involving them (Pearce and Sims, 2002). Again, this behavior may in some instances be adaptive, but is, overall, not in line with adaptive leadership behavior. While directive leadership assumes that the leader always knows the right way to act and should give commands accordingly, adaptive leaders rather try to provide orientation in a complex world and are willing to step back and let the team take the lead, whenever the situation calls for it. In summary, we propose that these divergent constructs are weakly related to adaptive leadership behavior:

H4: *Adaptive leadership behavior shows high construct validity by showing weak relations to the divergent constructs (a) laissez-fair leadership and (b) directive leadership.*

### Method

#### Sample and procedure

For the second study, data was again collected via the online data collection platform Prolific to reach a diverse sample. The requirements to take part in the study were the same as in Study 1 (i.e., being in an active employment relationship, having a direct supervisor and being fluent in English). The average response time was 20 min. As an incentive, participants received 2.32 pounds for their participation. Participation was voluntary and respondents were allowed to stop participation at any time.

In total, 345 participants completed the questionnaire. Due to too many missing values, 34 participants were excluded from the analysis, resulting in a final sample of 311 respondents. The sample comprised 200 females and 110 males, one person did not report their gender. The majority of respondents was between 24 and 35 years old (52.7%). Many participants (42.4%) worked for their current supervisor for 1–3 years, 25.1% for less than a year, 19.6% for 4–6 years and 12.9% for more than 6 years. Participants worked in a variety of branches, such as financial and business services, healthcare, civil services, engineering and consulting or IT.

#### Measures

Similar to the first study, all items were in English and rated on 5-point Likert type scales. For construct validity, we again used well-established scales and adapted some items to the business context and/or to the followers’ perspective. To measure authentic leadership behavior, we used the Authentic Leadership Inventory (ALI) by Neider and Schriesheim (2011). A sample item is “My supervisor shows consistency between his/her beliefs and actions.” Cronbach’s Alpha of this scale was 0.95 (95% CI [0.94, 0.95]). Transformational leadership was assessed the shortened form of the Multifactor Leadership Questionnaire (MLQ; Bass and Avolio, 1992) (Form 6S) to measure the four sub-dimensions of transformational leadership. A sample item is “My supervisor expresses with a few simple words what we could and should do.” Cronbach’s Alpha for transformational leadership was 0.95 (95% CI [0.95, 0.96]). We assessed servant leadership with the 28-item Servant Leadership Scale by Liden et al. (2015). A sample items is “My supervisor is interested in making sure that I achieve my career goals.” Cronbach’s Alpha was 0.97 (95% CI [0.96, 0.97]).

In Study 2, Cronbach’s Alpha for rigidity was 0.96 (95% CI [0.95, 0.97]). To further assess divergent validity, we used the respective items of the MLQ Form 6S (Bass and Avolio, 1992) to assess laissez-faire leadership. A sample item of laissez-faire leadership is “Whatever others want to do is O.K. with my supervisor.” Cronbach’s Alpha was 0.67 (95% CI [0.60, 0.73]). To measure directive leadership, we used six items of the Leader Behavior Items created by Pearce and Sims (2002). A sample item is “My supervisor gives me instructions about how to do my work.” Cronbach’s Alpha for this scale was 0.87 (95% CI [0.84, 0.89]).

### Results

In a first step, we conducted a PCA to analyze and confirm the factor structure of the selected 15 items based on Study 1. Results of parallel analysis, the scree plot and the initial eigenvalues (Component 1 = 10.17, Component 2 = 0.61) revealed a clear one-factor solution. The one-factor solution explained 67.80% of the variance and the scale showed high internal consistency (Cronbach’s Alpha = 0.97; 95% CI [0.96, 0.97]). All 15 items loaded strongly on this factor, with factor loadings ranging from 0.78 to 0.87 (see Table 2). Therefore, we decided to keep all 15 items in the scale.

**TABLE 2:** Results of Principal Component Analysis Study 2: Adaptive Leadership Behavior Scale (ALBS).

ALBS item	Component 1
1. My supervisor quickly grasps what kind of leadership behavior is optimal for a specific situation. (7)	0.81
2. My supervisor realizes when his/her leadership style should change due to changes in the situation. (8)	0.82
3. My supervisor tries to understand the needs of his/her subordinates and adjusts his/her responses in a fitting way. (11)	0.83
4. My supervisor recognizes changes in task priorities and the need to modify his or her leadership behavior. (13)	0.83
5. My supervisor is able to focus on and manage the task at hand while keeping an eye on employee’s needs. (5)	0.78
6. My supervisor is able to continuously adjust his/her behavior to the right degree to the circumstances at hand. (12)	0.87
7. My supervisor is capable of adjusting his/her leadership style based on the needs of his/her subordinates. (21)	0.87
8. My supervisor is able to balance opposite types of behavior (e.g., controlling vs. empowering) in a way that is appropriate for the situation. (24)	0.82
9. My supervisor is able to lead through difficulties, ambiguity and complexity. (25)	0.85
10. My supervisor is able to balance various conflicting needs of different stakeholders. (26)	0.82
11. My supervisor reacts to unforeseen circumstances or problems with an appropriate response. (15)	0.79
12. My supervisor adjusts his or her leadership behaviors to the demands of the specific situation (17)	0.84
13. My supervisor adapts his or her leadership behavior when unexpected events occur. (20)	0.81
14. My supervisor stays focused on the goal while remaining flexible in what leadership approaches, he/she uses to achieve the goal. (22)	0.83
15. My supervisor easily switches between directive and shared leadership according to the actual situation. (23)	0.78

*N* = 311. Results of Principle Component Analysis in Study 2 confirm the one-factor solution with 15 items. Corresponding item numbers of initial 27 item scale are displayed between brackets behind respective item.

In a next step, we assessed the model fit of the one-factor structure resulting from the PCA with a Confirmatory Factor Analysis (CFA) using Mplus 8, Version 1.8.6 (1). As our data did not follow a normal distribution, we used the conventional robust SE estimator (MLM) as estimation technique (Lai, 2018). We used different fit indices to assess model fit, such as the chi-square test of model fit (χ^2^), comparative fit index (CFI), root-mean-square error of approximation (RMSEA) and standardized root-mean-square residual (SRMR). The result of the chi-square test was χ^2^ (*df* = 105, *N* = 311) = 2770.571 (*p* < 0.001), suggesting that the fit of the data to the hypothesized model is not perfect. However, the chi-square test is known as a very sensitive fit index, especially to the sample size, and therefore other fit indices are analyzed as well (Byrne, 2013). The one-factor model yielded an acceptable fit according to CFI (0.98) and SRMR (0.03) values. With a RMSEA estimate of 0.04 (95% CI [0.03, 0.06]; RMSEA *p*-value < 0.817), the RMSEA suggested a moderate fit. The standardized factor loadings ranged from 0.76 to 0.87 (see Supplementary Table 1). In summary, our results confirm an acceptable fit to the one-factor solution to the data.

Lastly, we computed and compared omega-hierarchical values for the general factor adaptive leadership (ωH = 0.97) as well as for a general factor of adaptive leadership behavior with four sub-factors relating to the four aspects of adaptive leadership behavior (ωH = 0.96). Both results further support the unidimensionality of the scale.

#### Construct validity

In line with Hypothesis 3, adaptive leadership behavior correlated positively with additional convergent constructs, namely authentic leadership (*r* = 0.84, *p* < 0.001), transformational leadership (*r* = 0.81, *p* < 0.001), and servant leadership (*r* = 0.79 *p* < 0.001). Supporting Hypothesis 4, rigidity was again strongly negatively correlated (*r* = −0.66, *p* < 0.001) to adaptive leadership. Directive leadership (*r* = 0.28, *p* < 0.001) and laissez-faire leadership (*r* = 0.29, *p* < 0.001) showed moderate correlations to adaptive leadership behavior, thus lending tentative support for Hypothesis 4.

#### Common method bias

Similarly to Study 1, we again tested the relationship between adaptive leadership and both convergent and divergent constructs for common method effects (Podsakoff et al., 2003). The relationship of adaptive leadership with authentic leadership (estimate = 0.73, *p* < 0.001), transformational leadership (estimate = 0.68, *p* < 0.001), as well as servant leadership (estimate = 0.62, *p* < 0.001) remained significant after controlling for a method factor (i.e., by using conscientiousness as unrelated variable to the research question of Study 2 but collected with the same methodology). Also, the relationship between adaptive leadership and rigidity still showed a significant, negative relationship (estimate = −0.38, *p* < 0.001). Similarly, the relationship between adaptive leadership and directive leadership (estimate = 0.12, *p* = 0.024) or laissez-faire leadership (estimate = 0.24, *p* < 0.001) remained significant after correcting for common method bias.

## Study 3

In Study 3, our goal was to confirm the model fit of the one-factor structure of the ALBS with an independent sample using CFA and additionally assess its criterion-related validity. To assess criterion-related validity, we included different outcome variables that have been used for previous scale validations in leadership research (e.g., Kalshoven et al., 2011) and/or that seemed to be relevant for adaptive leadership behavior. Thus, we included perceived leader effectiveness (follower rated), job satisfaction (follower rated), perceived employee effectiveness (leader rated) and an indicator of leader’s wellbeing, irritability (leader rated), as criteria in our study.

Adaptive leaders do not only react to upcoming situational demands but also adapt to the daily needs, experiences or skill levels of their employees (Yukl and Mahsud, 2010). When employees feel that their leader understands and truly cares for their individual needs and adapts their behavior according to the situation, followers’ job satisfaction and their perception of leadership effectiveness should be higher as well. Similarly, by truly understanding a follower’s needs and the situation at hand, leadership behavior can be adapted more specifically to those needs and hence support the employee in the best possible way, thus, increasing employee effectiveness.

In addition to positive outcomes for followers, adaptive leadership may also benefit leaders themselves. We propose that by adjusting their leadership behavior to the demands of a specific situation, a leader’s wellbeing is enhanced (e.g., is related to lower cognitive and emotional irritation). This assumption is based on fit theory that proposes that people thrive to fit the environment because they aim for a maximum consistency among the environment as well as both own and other people’s behaviors (Vianen, 2018).

Summed up, we propose that adaptive leadership behavior contributes positively to followers’ job satisfaction, leader and follower effectiveness and higher leader wellbeing (i.e., less irritation) by flexibly adjusting to what employees or situational demands require.

*H5: Adaptive leadership behavior is positively related to (a) follower’s job satisfaction, (b) followers’ perceived leader effectiveness, (c) leaders’ perceived employee effectiveness and (d) leaders’ wellbeing.*

### Method

#### Sample and procedure

Study 3 was a cross-sectional multi-source study with 155 leader-follower dyads in Germany. We recruited dyads via social media platforms such as LinkedIn, Xing or Facebook, by directly approaching employees of multiple organizations or via personal networks. Participants did not receive any incentive besides a summary report of the overall research results. The only inclusion criteria that we applied were being in an active employment relationship, having a direct supervisor or follower as well as being fluent in German.

Overall, 245 leader-follower dyads were initially registered to take part in the study, of which 197 leaders and 218 followers filled in the survey. Participants whose partners did not complete the questionnaire or who discontinued to fill in the survey themselves were excluded from the analysis, resulting in a final sample of 155 complete dyads. The average age of leaders was 44 years (*M* = 44.2, *SD* = 11.2). On average, they were responsible for 21 employees (*M* = 20.7, *SD* = 57.6) and worked 45 h per week (*M* = 45.0, *SD* = 11.8).

On average, employees were 35 years old (*M* = 34.6, *SD* = 11.7) and worked for 15 years (*M* = 14.7, *SD* = 12.7). Many of the participants (41.3%) worked for five or more years together with their current leader (*M* = 5.7, *SD* = 6.2). The majority of participants (73.5%) stated that they worked together with their leader on a daily basis, 18.1% on a weekly basis and 8.3% saw their supervisor once a month or less.

#### Measures

As data collection took place in Germany, we translated the English items into German following the guidelines by Brislin (1970) if no German version of a scale was available. In that case, one bilingual person was briefly introduced to the concepts and translated the original questionnaire from English to German. Next, the German version was back-translated to English by another, independent bilingual translator. This final translation was then jointly discussed between the native speakers to reach consensus and make adjustments to the German version if necessary. The translation process was reviewed afterward to make sure that the content and meaning of the translated version remained unchanged.

All items except the one for job satisfaction were assessed on a Likert type scale ranging from 1 (*strongly disagree*) to 5 (*strongly agree*). To measure perceived employee effectiveness, we used two items from Kalshoven et al. (2011). The first item is “How effective is the employee in his/her daily work?” and the second item is “To what extent is the overall functioning of the employee satisfactory?” Perceived leader effectiveness was measured with four items developed by Bass and Avolio (1995) and translated by Felfe (2006). A sample item is “My supervisor ensures satisfaction through his/her leadership behavior.” Job satisfaction was measured with the item “How satisfied are you with your work in general?” using a 5-point Kunin-scale (Wanous et al., 1997; Franke and Felfe, 2008). Leader’s irritability was measured with the irritation scale by Mohr et al. (2007). Three items measure cognitive irritation, an indicator of job-specific stress (e.g., “Even at home I often think of my problems at work”) while four items measure emotional irritation, an indicator of social stress (e.g., “I get grumpy when others approach me”).

### Results

Results of the CFA for the one-factor solution of the ALBS showed that the data fits the one-factor model well. Descriptive statistics and correlations are depicted in Table 3. For this sample, fit indices were: χ^2^ (*df* = 105, *N* = 155) = 1401.303 (*p* < 0.001), CFI = 0.94, RMSEA = 0.07 (95% CI [0.06, 0.09], RMSEA *p*-value = 0.02), SRMR = 0.04. The standardized factor loadings ranged from 0.64 to 0.85 (see Supplementary Table 2). Most, but not all, factor loadings are comparable to those of Study 2.^2^

**TABLE 3:** Descriptive statistics and correlations for variables in Study 3.

Variable	*M*	*SD*	1	2	3	4	5	6	7	8	9	10
1. Follower Age	34.55	11.67	(–)									
2. Follower Gender^a^	1.54	0.49	0.02	(–)								
3. Leader Age	44.23	11.16	0.42**	−0.05	(–)							
4. Leader Gender^a^	1.32	0.47	−0.10	0.30**	−0.27**	(–)						
5. Lengths of relationship (Dyad)	5.68	6.21	0.52**	0.10	0.43**	0.00	(–)					
6. Adaptive Leadership Behavior (FR)	3.80	0.68	0.03	−0.00	−0.03	0.03	−0.11	(0.95)				
7. Job Satisfaction (FR)	4.12	0.65	−0.01	−0.14^†^	−0.03	−0.08	−0.14	0.16*	(–)			
8. Perc. Leader Effectiveness (FR)	4.05	0.68	0.01	−0.08	−0.11	0.01	−0.18^†^	0.78**	0.33**	(0.84)		
9. Perc. Employee Effectiveness (LR)	4.30	0.55	−0.03	0.09	0.05	−0.02	0.02	0.08	0.10	0.14^†^	(0.80)	
10. Leader’s Irritability (LR)	2.36	0.68	−0.02	0.02	−0.16^†^	0.01	0.00	−0.14^†^	0.06	−0.10	−0.11	(0.82)

*N* = 155, *p < 0.05.

**p < 0.01. ^†^<0.10. ^a^Gender: 1 = male, 2 = female, 3 = diverse; FR, Follower Rating; LR, Leader Rating.

Cronbach’s coefficient alpha is displayed on the diagonal.

#### Criterion-related validity

Results showed that adaptive leadership behavior and follower’s job satisfaction were significantly related (*r* = 0.16, *p* = 0.050), supporting Hypothesis 5a.

In addition, adaptive leadership behavior showed a high positive correlation with perceived leader effectiveness (*r* = 0.78, *p* < 0.001), supporting Hypothesis 5b. However, the results did not show a significant relation between adaptive leadership behavior and perceived employee effectiveness (*r* = 0.08, *p* = 0.304), thus Hypothesis 5c was not supported. Finally, adaptive leadership behavior was only marginally related to irritability (*r* = −0.14, *p* = 0.107), thus, tentatively supporting Hypothesis 5d.

#### Supplementary analysis

Our findings did not support Hypothesis 5c, that adaptive leadership behavior is positively related to a higher perception of employee effectiveness. However, we wanted to explore further if the length of the leader-follower working relationship impacts this relationship. We suspected that the time leaders and their followers have been working together might moderate the relationship between adaptive leadership behavior and leaders’ perceived employee effectiveness. Results showed that the interaction effect between adaptive leadership behavior and length of the working relationship on perceived employee effectiveness was significant. While the relationship between adaptive leadership and leaders’ perceived employee effectiveness was significant when leaders worked with their follower for a longer amount of time (*B* = 0.16, *p* < = 0.016), it was not significant when leaders had worked with their follower for a shorter amount of time (*B* = −0.09, *p* < = 0.173) (see Figure 1).

## General discussion

Adaptive leadership is a construct that has received considerable attention in the past years. Its important role for organizational functioning in today’s VUCA world is undisputed. However, the concept still needs further refinement, tangibility and empirical scrutiny (Yukl and Mahsud, 2010). Therefore, the purpose of our study was to develop a concise, behavior-oriented instrument for adaptive leadership and establish empirical support for its relevance in today’s workplaces. We validated this newly developed instrument with three independent data sets in order to determine its psychometric properties as well as evidence for both construct (i.e., convergent and divergent validity) and criterion-related validity. Establishing a new measure for adaptive leadership is important as it builds the ground for further empirical research on the role and impact of adaptive leadership in organizations as well as for developing concrete action points for leadership programs and interventions.

### Construct validity

Based on a thorough literature review, we defined four defining behaviors that constitute the construct of adaptive leadership. Results of all PCAs show a clear one-factor solution, hence the four behaviors do not seem to represent distinct factors but rather highly interrelated facets of the same one-dimensional construct. The fit indices of the CFA attest an acceptable fit to the data, supporting the one-factor solution.

In addition, we found positive correlations among the ALBS and proposed convergent constructs such as cognitive flexibility, emotional intelligence, authentic leadership, transformational leadership and servant leadership. Thus, the ALBS relates to constructs that share a certain conceptual overlap although being sufficiently distinct. Also, we were able to show discriminant validity as adaptive leadership behavior had negative or no significant relationships to divergent constructs such as rigidity and laissez-faire leadership and directive leadership, respectively.

After correcting for common method bias, results showed a decrease in the estimated strength of relationships between adaptive leadership and convergent as well as divergent constructs. However, for both convergent and divergent constructs, the relationships still remained significant. This suggests that the estimated strength of relationships might have been inflated to some extent, due to the common method used to assess the construct variables (i.e., by means of a self-report survey). It must be noted, however, that this likely also applies to the reported relationships for convergent/divergent constructs in other scale validation studies (in the field of leadership).

### Criterion-related validity

Our results show support for criterion-related validity of the ALBS. In line with previous research, we decided to select three outcome variables that have already been used in other leadership scale development and validation papers (e.g., Brown et al., 2005; Kalshoven et al., 2011). As proposed, we found significant positive relationships between adaptive leadership behavior and perceived leadership effectiveness as well as follower’s job satisfaction. Thus, the more adaptive leadership behavior is shown, the more effective do followers perceive their supervisor’s leadership behavior. Also, the more adaptability the supervisor shows in their leadership behavior, the higher the followers’ job satisfaction. Both outcomes as well as adaptive leadership behavior have been assessed by followers. Hence, common source bias might have affected these results (Podsakoff et al., 2003; Spector, 2019). Therefore, we also included outcome variables that were rated by the leader such as perceived employee effectiveness and leader’s irritability. As results show, the effect of adaptive leadership behavior on perceived employee effectiveness was not significant. As a supplementary analysis revealed, however, when leader and follower had been working together for a longer time, adaptive leadership was significantly related to leaders’ perception of their employee’s effectiveness. One explanation may be that the longer leaders know their employee, the better they understand and anticipate their needs, thus, being better able to adapt accordingly. When leaders adapt their behavior to the followers’ needs, employees are supported in the best possible way and, consequently, are able to perform more effectively (Meglino, 1998; De Vries and Florent-Treacy, 2002; Oh et al., 2020). Although this *post hoc* explanation could be supported in our study, future research should confirm this finding with additional samples. Finally, we extended previous research with a less common outcome variable in scale development papers as it seemed to be a relevant outcome variable of adaptive leadership behavior in dynamic environments. Our findings tentatively supported our assumption that more adaptive leadership behavior is related to lower levels of leaders’ irritability. Hence, it seems that adaptive leadership behavior has a positive effect on the wellbeing of leaders themselves. This is not surprising as work strain usually results from the interplay of personal and environmental characteristic (Huang and Simha, 2018). Once a leader acts in congruence with the needs of the environment and those of the employees, positive outcomes as well as psychological wellbeing may result (Edwards et al., 1998; Lee and Antonakis, 2014). In summary, the ALBS shows good criterion-related validity. Future research may build on these findings and test further outcome variables of adaptive leadership behavior to support its important role for organizational functioning.

### Strengths, limitations and suggestions for future research

The current study has several strengths. The newly developed instrument has been developed based on an extensive literature review and was validated with three independent, diverse data sets that each had a relatively large sample size. For the whole scale development and validation process, we followed the recommended steps by Hinkin (1998) and assessed both construct as well as criterion-related validity. The factor structure and model fit was re-tested and confirmed in independent samples (Hinkin, 1998). To assess criterion-related validity, we did not only include follower ratings but also ratings from leaders themselves (e.g., relating adaptive leadership behavior rated by followers and leader’s irritability rated by leaders) to reduce common source effects (Podsakoff et al., 2003).

Nevertheless, there are also limitations and recommendations for future research. Scale development is a continuous process and this paper only represents an initial step in the validation process of the ALBS. Additional research is needed to further assess the validity of the newly developed instrument within different contexts and cultures. Also, this research relies on subjective ratings of leader’s or follower’s rather than on objective performance measures which is a well-known limitation of survey research (Kaiser et al., 2008; Yukl, 2010). Especially in Study 1 and 2, common source effects might have inflated the results as we relied exclusively on single source ratings here. However, when making this decision, we carefully considered what would be the most suitable perspective for an accurate assessment of the observed variables in our initial studies. Since followers are the recipients of leadership behavior, it is logical that their perspective allows them to assess it best. In Study 3, we included both self- and other-ratings, to circumvent potential common source effects and investigate the criterion-related validity with different sources. Future research should use a multi-source design to extend the present study. From our perspective, it might be very interesting to see, for example, how self- and other-ratings differ in regard to adaptive leadership behavior. It might be that leaders provide more accurate or comprehensive ratings of their adaptive behavior because they are also able to rate their internal thoughts on their behavioral strategy selection. A comparison of both self- and other ratings might shed further light on this aspect.

Furthermore, the data of this study is assessed in a cross-sectional way. Cross-sectional designs do not allow any inference on causality. To account for this limitation, future research could conduct longitudinal studies to observe adaptive leadership behavior over a longer period of time. This would also allow to examine adaptive leadership behavior across changing situations which is most suitable when we consider that adaptive leadership is required in a dynamic environment (Yukl and Mahsud, 2010). As one example, future research could assess adaptive leadership in a diary study, testing whether adaptive leadership behavior fluctuates across situations. Previous leadership research emphasizes that a within-person approach is the most suitable way to research the dynamic aspects of leadership behavior (Breevaart et al., 2016). With a diary design, it is possible to analyze, for example, which circumstances allow leaders to execute adaptive leadership behavior or how fluctuations in adaptive leadership behavior influence the daily work of employees. These insights would advance our understanding for situational predictors of and contextual boundary conditions for adaptive leadership behavior and its effectiveness (Yukl and Mahsud, 2010). In addition to situational antecedents, also personal antecedents of adaptive leadership as well as potential mechanisms could be investigated in the future. As shown in our study and indicated by past research, a leader’s emotional intelligence could play an important role as a personal antecedent in how well a leader is able to assess the situation and employee’s needs, to react flexibly to those situational needs and to, ultimately, lead adaptively (Yukl and Mahsud, 2010).

### Practical implications

The development of the ALBS has not only important implications for future research but also for practice. Gaining a deeper understand of concrete leadership behaviors that are key in VUCA environments is extremely valuable for today’s organizations. The four proposed aspects of adaptive leadership behavior may guide practitioners in designing training interventions to support a leader’s ability to assess the needs of the situation (i.e., environmental and employees’ needs) and to flexibly select adequate leadership behaviors accordingly. With the newly developed ALBS, we offer organizations a reliable and valid instrument to examine their leaders’ adaptive leadership behavior. Furthermore, the relationships of the ALBS to a variety of work outcomes emphasizes the impact that adaptive leadership can have on both leaders’ and follower’s performance and wellbeing. Due to the complex and fast-paced environment that the business world is facing today, the topic of adaptive leadership behavior is particularly timely and relevant for organizations.

## Data availability statement

The raw data supporting the conclusions of this article will be made available by the authors, without undue reservation.

## Ethics statement

The studies involving humans were approved by the Ethics Review Committee Psychology and Neuroscience (ERCPN)–Maastricht University. The studies were conducted in accordance with the local legislation and institutional requirements. The participants provided their written informed consent to participate in this study.

## Author contributions

SN, AN, and SU contributed to the conception and design of the study. SN was mainly responsible for the whole manuscript including data collection, statistical analysis, and wrote the first draft of the manuscript. SN was supported by AN during the whole process. JS supported critical points in the statistical analysis. AN, SU, and UH gave regular feedback and discussed critical points. All authors contributed to manuscript revision and read and approved the submitted version.

## Footnotes

1
https://www.prolific.co/

2 Deviations might be due to differences in sample characteristics. Compared to a more diverse, English-speaking sample in Study 2, the sample in Study 3 was more homogenous with only German-speaking participants. Thus, cultural differences might have caused the differences between factor loadings. In addition, although we followed the recommended procedure by Brislin (1970) or used already well-established scale translations from previous studies, differences in factor loadings between Study 2 and 3 could also be due to the translation issues.
