# Supplementary material for: Development and validation of the adaptive leadership behavior scale (ALBS)
Source: Front Psychol. 2023 Sep 27;14:1149371. doi: 10.3389/fpsyg.2023.1149371 (PMC10565815; doi:10.3389/fpsyg.2023.1149371)
Supplement: Supplementary file 2 [file Table_2.docx]

**Appendix/Supplementary Material**

**Supplementary Table 2**

*Results of Confirmatory Factor Analysis Study 3: Adaptive Leadership Behavior Scale (ALBS)*

| ALBS item | Factor Loading | 95% CI |
| --- | --- | --- |
| 1. My supervisor quickly grasps what kind of leadership behavior is optimal for a specific situation. (7) | .81 | .76; .87 |
| 1. My supervisor realizes when his/her leadership style should change due to changes in the situation. (8) | .78 | .69; 87 |
| 1. My supervisor tries to understand the needs of his/her subordinates and adjusts his/her responses in a fitting way. (11) | .69 | .58; .80 |
| 1. My supervisor recognizes changes in task priorities and the need to modify his or her leadership behavior. (13) | .81 | .74; .88 |
| 1. My supervisor is able to focus on and manage the task at hand while keeping an eye on employee’s needs. (5) | .74 | .65; .83 |
| 1. My supervisor is able to continuously adjust his/her behavior to the right degree to the circumstances at hand. (12) | .85 | .81; .89 |
| 1. My supervisor is capable of adjusting his/her leadership style based on the needs of his/her subordinates. (21) | .81 | .72; .89 |
| 1. My supervisor is able to balance opposite types of behavior (e.g., controlling vs. empowering) in a way that is appropriate for the situation. (24) | .80 | .74; 87 |
| 1. My supervisor is able to lead through difficulties, ambiguity and complexity. (25) | .70 | .60; 80 |
| 1. My supervisor is able to balance various conflicting needs of different stakeholders. (26) | .64 | .54; .75 |
| 1. My supervisor reacts to unforeseen circumstances or problems with an appropriate response. (15) | .70 | .61; .79 |
| 1. My supervisor adjusts his or her leadership behaviors to the demands of the specific situation. (17) | .80 | .72; .88 |
| 1. My supervisor adapts his or her leadership behavior when unexpected events occur. (20) | .82 | .74; .89 |
| 1. My supervisor stays focused on the goal while remaining flexible in what leadership approaches, he/she uses to achieve the goal. (22) | .76 | .68; .84 |
| 1. My supervisor easily switches between directive and shared leadership according to the actual situation. (23) | .70 | .60; .80 |

*Note.* *N* = 155. Standardized factor loadings of Study 3 are displayed with their corresponding 95% confidence intervals.
